# Automated tracking of broiler breeder activity and functional area use in a commercial housing system: differences between sexes and time of day

**DOI:** 10.1016/j.psj.2025.106304

**Published:** 2025-12-18

**Authors:** Malou van der Sluis, Annemarie J.W. Mens, Rick A. van Emous

**Affiliations:** a Animal Breeding and Genomics, Wageningen University & Research, 6700 AH Wageningen, the Netherlands; b Animal Nutrition, Wageningen University & Research, 6700 AH Wageningen, the Netherlands

**Keywords:** Automation, Ultra-wideband, Behaviour, Poultry

## Abstract

Broiler breeders are in the EU traditionally housed in large flocks, yet little information is available regarding the birds’ movement patterns in these flocks and their use of functional areas within the house (e.g., nest boxes, litter area). This exploratory study aimed to gain some first insights into individual differences in spatial area use of broiler breeders under commercial conditions, using an ultra-wideband (**UWB**) tracking system. The broiler breeder house (59 × 14 m; 826 m^2^) under study housed 5,600 Ross 308 breeder females and 350 Ross 308 breeder males and consisted of two compartments that the birds could not move between, each containing three functional zones: a litter area, an elevated slatted area, and a nest box area. A small subset of birds was fitted with a backpack containing an UWB tag, and data on 7 male (**M**) and 8 female (**F**) birds from 52 to 55 weeks of age were examined here. Four aspects of activity and functional area use were investigated: 1) overall area use, 2) distances moved, 3) relative time spent in functional areas, and 4) consistency in resting locations at night. On average, broiler breeders used more than half of the area available to them within a day, although this varied between individuals and/or days. In general, M birds covered larger distances in a day than F birds, and distances moved were larger during the light period (04:00-17:00) and transition period (17:00-17:30) than during the dark period (17:30-04:00). Furthermore, M birds spent less time in the nest and slats zones, and more time in the litter zone, than F birds. No differences between the sexes were observed in the average distance between resting locations at night. Although the sample size was very limited and these findings should therefore be considered as preliminary, they do offer a first step towards providing insight into broiler breeders’ movement patterns and preferred locations, which can in the future inform housing design and management.

## Introduction

Broiler breeders are traditionally housed in systems with both litter and slatted areas, typically in flocks ranging from 3,000 to 10,000 birds. Given these large flock sizes, assessments of behaviour and welfare often focus on the group as a whole rather than on individual birds. Consequently, limited information is available regarding the birds’ movement patterns and use of functional areas within the house, such as nest boxes, roosting places, and feeding and drinking lines.

In the 1980s, the movement of individually identified broiler breeders in two flocks of nearly 4,000 birds each, kept in deep litter houses (46 × 15 m), was studied (Appleby et al., 1985). In the first flock, 25 female birds were marked with numbered wing tags, while in the second flock 25 male and 25 female birds were marked. It was observed that individual breeders used more than half of the house, with a median coverage of 73% (ranging from 56% to 95%) in the first flock. In the second flock, movement patterns of males and females were recorded on 10 days across 7 weeks. This revealed no significant differences between the sexes in their ranges.

In a recent, unpublished study conducted by our lab (van Emous, unpublished data) on a commercial broiler breeder farm (house dimensions 85 × 12 m, with 50% central slatted area, housing approximately 7,000 breeders), individual movement patterns were also investigated. The house was divided into 44 zones (each 8 × 3 m), and nine females and ten males were individually marked with colored stripes or dots. Their positions were visually recorded over a four-week period, during a photoperiod of 13 h. Preliminary observations indicated considerable individual variation in space use, with individuals occupying between 15% and 93% of the available house area. On average, males utilized a larger proportion of the house (76%) compared to females (60%) and were more frequently found on littered areas (76% versus 46% for females). Contrasting with the results from Appleby et al. (1985), this suggests potential sex differences in space and functional area use in broiler breeder houses.

For broilers, large variation in movement patterns has been observed in a commercial broiler house, using a Real Time Location System based on ultra-wideband (**UWB**) technology (Baxter and O'Connell, 2023). The UWB system provided XY coordinates of individual broilers with an accuracy of less than 30 cm and offered a highly precise method for studying spatial behaviour in commercial housing conditions over longer periods of time. Using the resulting UWB data, Baxter and O’Connell (2023) found no consistent evidence that broilers established similarly sized preferred areas. Some individuals remained within 10 meters of their original location for most of the observation period, whereas others explored at least 90% of the available house area. Given the large amount of individual-level location data that could be obtained using this UWB system, a similar approach could help to provide more insight into broiler breeder movement and area use.

A better understanding of the movement patterns and space use of broiler breeders in commercial housing systems is essential for improving both welfare and reproductive performance, by designing breeder houses that effectively accommodate the (potentially varying) needs of the individual birds. Therefore, the aim of this exploratory study was to gain initial insights into individual differences in spatial use and functional area preferences of broiler breeders under commercial conditions, using an UWB tracking system. Given the limited sample size, the findings should be considered as preliminary, offering a first step towards more detailed future investigations.

## Materials and methods

### Ethical statement

All procedures involving animals were conducted in accordance with Dutch legislation and institutional guidelines for the ethical use of animals in research. The study was observational in nature and carried out on a commercial broiler breeder farm. No invasive procedures were performed, and care was taken to minimize disturbance to the animals. The tagging method was non-invasive and involved the use of lightweight backpacks well within the generally accepted threshold of maximum 5% of animals’ body weight in animal tracking studies. The research protocol was reviewed by the Animal Welfare Body of Wageningen Research (IvD-WR) and classified as not constituting an animal experiment under the European Directive 2010/63/EU on the protection of animals used for scientific purposes.

### Housing and birds

**Fig. 1:**
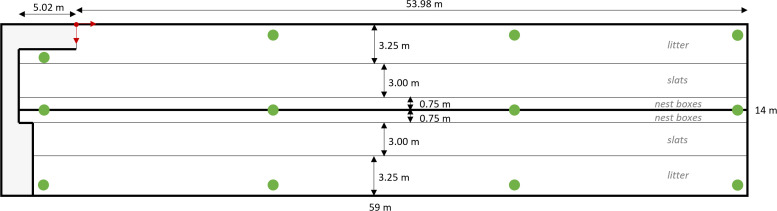
**Schematic representation of the functional zones in the broiler breeder house.** Green dots indicate the position of the UWB anchors in the house.

The study was conducted in July 2024 on a commercial broiler breeder farm with five breeder houses located in The Netherlands. One house, measuring 59 × 14 meters (826 m²), was equipped with an UWB tracking system and housed 5,600 Ross 308 breeder females and 350 Ross 308 breeder males during the observation period (52–55 weeks of age). The house followed a standard EU layout, featuring a central slatted area with a community nest situated above it. It was divided into two compartments along the length of the house (left and right), each containing three functional zones (Fig. 1): a litter area (3.25 m wide), an elevated slatted area (3.0 m wide), and a nest box area (0.75 m wide). Due to the physical separation of the house, birds could only access the compartment in which they were placed (left or right side). The litter area was covered with a mixture of wood shavings and dry manure up to 10 cm deep. Feed was provided via six female feeder lines (Bridomat™, Roxell, Belgium), of which four were located on the slats and two on the litter. The female-feeder lines were equipped with adjustable roller tubes to prevent males from accessing the female feeders. Additionally, two male Bridomat™ feeder lines were installed at a minimum height of 50 cm above the litter, to prevent female birds from eating from the male feeders, with one on each side of the house. Feed was provided immediately after lights on (i.e., at 04:00 h). Water was supplied via 70 bell drinkers (35 per side) positioned on the slats approximately 50 cm from the nest boxes. The breeders were kept on a photoperiod of 13 h of light (light period, **LP**) and 11 h of darkness (dark period, **DP**) (13L:11D), with lights on from 04:00 to 17:00 h. A transition period (**TP**) from 17:00 to 17:30 h, during which light intensity gradually decreased, was implemented to resemble a more natural day-night transition and encourage birds to move onto the slatted area. There was no transition period for lights on in the morning.

### Ultra-wideband tracking system

The breeder house was equipped with a commercially available UWB tracking system, enabling continuous real-time tracking of individually tagged breeders at approximately one-second intervals for 24 h per day, and the resulting data of 22 days (from 52 to 55 weeks of age of the broiler breeders) were used. The UWB system was manufactured by SEWIO (Brno, Czech Republic) and supplied by Noldus Information Technology (Wageningen, The Netherlands). A total of 12 UWB anchors were installed in the house, arranged in three rows of four anchors each: one row along the left wall, one row above the central nest boxes, and one row along the right wall (Fig. 1). All anchors were mounted at a height of 230 cm above the floor.

Twenty individual breeders (4 males (**M**) and 6 females (**F**) per side; randomly selected but only including healthy birds) were equipped with khaki backpacks (Hen Apron, Cikonielf, China). Each backpack measured approximately 20 × 20 cm and contained a sewn-on pouch, secured with a tie wrap, that held the UWB tag (45 × 54 mm; 28 g). The backpacks were positioned on the birds’ backs using 3-mm wide elastic wing straps. The combined weight of the backpack and tag was 62 g, corresponding to 1.2% of the males’ average body weight (5.0 kg) and 1.6% of the females’ average body weight (4.0 kg), which is well below the recommended threshold of 5% additional body weight for tracking devices used in wildlife studies (Kenward, 2001). During the study, 5 broiler breeders lost their backpacks, leaving 4 M and 3 F birds with UWB tags on the left side, and 3 M and 5 F birds with UWB tags on the right side of the house. No broiler breeders without backpacks were studied (i.e., there was no control group for the backpacks), as other studies have shown limited impact of backpacks on broilers (Stadig et al., 2018b; Baxter and O’Connell, 2020).

### Determining area use

**Fig. 2:**
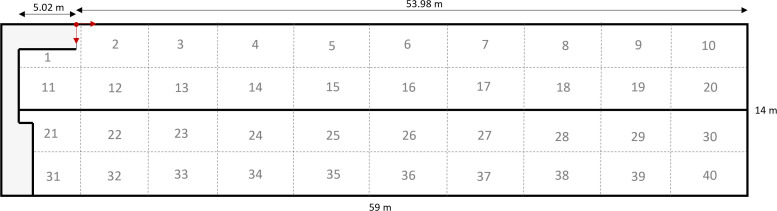
**Visualization of the virtual zones that were assigned to assess area use of the birds.** Each zone is 3.5 × 5.9 m, with the exception of zones 1, 11, 21 and 31.

To determine area use in the tagged broiler breeders, the area available to the birds was virtually split into 40 zones (Fig. 2; similar to the approach of Baxter & O’Connell (2023)). Each zone had a size of 3.5 × 5.9 m (20.65 m^2^ each), with exception of the zones on the far-left side of the house, i.e., zones 1, 11, 21 and 31, due to access to the house being located there. It could occur that a bird was detected in the wrong section of the house, due to noise in the UWB detection. This was the case for approximately 0.015% of all detections (see **Supplementary Data 1**) and these instances were subsequently filtered out. Moreover, due to noise in the UWB detections birds were sometimes registered outside the predetermined house dimensions. In these instances (1.78% of the recorded observations; registered out of bounds in the x-direction or y-direction or counted twice if out of bounds in both directions), the recorded locations were linked to the closest connecting zone in the y-direction for erroneous y-detections and in the x-direction for erroneous x-detections, as these were considered to be the most likely zones that the birds were truly located in. Subsequently, it was summed per bird and day how many different zones were visited. The number of zones visited per bird per day was used for further statistical analysis.

### Assessing distances moved

To determine distances moved by the broiler breeders, the Euclidian distance between subsequent detections of the same birds was calculated. It is important to note that no filtering to exclude potential noise in the data (i.e., very small movements due to position estimation errors) was implemented here, which could have resulted in a substantial overestimation of distances moved due to the noise adding up over time (see Discussion for an elaborate discussion of the potential impact of this noise). The distance calculations were made per day (total daily distance moved) and per light/dark period (light period, dark period and transition period), where distances moved per hour were calculated within the periods to account for different durations of the periods within a day, resulting in distances moved being expressed in meters per hour. To determine whether distance moved and area use were correlated, the association between distances moved and area used were assessed using correlation analysis (see section Statistical Analyses).

### Determining time spent in functional zones

To determine the time spent in functional zones, the house was divided into three sections per side: the litter area (**L**), the slats (**S**), and the nest boxes (**N**), as shown in Fig. 1, and it was determined in which functional zone birds were located based on their recorded UWB positions. Detections on the wrong side of the pen (due to noise in the UWB data) were excluded. It was summed per bird and day or period how much time (based on the number of detections in a zone as a percentage of the total number of detections) was spent in the different functional zones. It is important to note that the nest boxes were closed for most of the dark period but opened at 02.00 h in the morning and closed at 16.00 h.

### Determining resting location at night

To determine how consistent the broiler breeders were in where they spent their time at night, the last location detection before lights on (i.e., around 04:00 h) and the first location detection after lights fully off (i.e., after the transition period, around 17:30 h) were determined per bird and day. Birds can also move in the dark – and thus change location during the night – but this was considered to be the best indicator of whether birds would return (at night) to where they started the day (at the onset of the light period). The Euclidean distance between these two points was calculated for further examination.

### Statistical analyses

All statistics were performed in R version 4.4.1 (R Core Team, 2024). To study the number of zones visited by the birds over time, linear mixed effects models were implemented using the lme4 (Bates et al., 2015) and lmerTest (Kuznetsova et al., 2017) packages, with sex of the bird, side of the pen and day of recording (i.e., 22 days) as fixed effects, and a random effect for the intercept of birds, to take into account repeated measurements on the same birds. The models were fitted for full day data, and for the three periods (light, dark and transition period) separately. To study the daily distance moved by the birds, linear mixed effects models were again implemented, with sex of the bird, side of the pen and day of recording as fixed effects, and a random effect for the intercept of birds. A similar model but including period as a fixed effect as well as the interaction between period and sex of the birds, was implemented to assess average distances moved per hour in the different periods. Subsequently, contrasts were determined using the emmeans package (Lenth, 2024), with a sidak p-value adjustment for multiple comparisons. The correlation between distance moved and area use in a day was determined using Kendall’s tau correlation, as the number of zones visited was considered ordinal data (albeit included as numeric in the correlation calculation) and there were many ties in the data (i.e., data points with the same value for number of zones visited). To assess the proportion of time spent in different functional zones, beta binomial models were implemented using the glmmTMB package (Brooks et al., 2017) to account for the count data and the constraints in the underlying proportional data (i.e., proportions always add up to 1 and cannot be lower than 0). The model included day of recording and an interaction between sex of the birds and the functional zone as fixed effects, and a random effect for the intercept of the birds to account for repeated measurements on the same birds. To determine differences between the different levels of sex and functional zone, contrasts were determined using the emmeans package (Lenth, 2024), with a sidak p-value adjustment for multiple comparisons. Subsequently, the proportion of time spent in different functional zones per period (light period, dark period and transition period) was determined using three separate beta binomial models (one per period), with the same fixed and random effects as for the model for full days. To study the distance between subsequent night resting spots, a linear mixed effects model was again implemented, with sex of the bird, side of the pen and day of recording as fixed effects, and a random effect for the intercept of birds. The ggplot2 (Wickham, 2016) and ggpattern (FC et al., 2024) packages were used to make the visualizations. The level of statistical significance was set at 0.05.

## Results

### Descriptive statistics

Table 1 shows the mean, minimum and maximum observed values for the area use, distances moved, and time spent in functional zones, for the two sexes and the different light periods. The mean distance between resting locations at night was 9.2 m (SD 7.4 m) for males (range: 0.4-43.1 m) and 8.8 m (SD 6.2 m) for females (range 0.3-27.2 m).

**Table 1:** The mean (SD) and range [minimum - maximum] of observed values for the area use, distances moved, and time spent in functional zones, for the two sexes and the different light periods, per day. *L* = litter; *S* = slats, *N* = nest boxes.

	Area use (# zones)	Distance moved (m)	Time spent in functional zones (% of time)
*Full days*			
Males	14.2 (4.2) [6-20]	3131 (544) [1898-4965]	L: 58 (25) [18-100] S: 43 (24) [0-82] N: 0 (2) [0-16]
Females	11.5 (4.3) [3-20]	2283 (556) [360-3804]	L: 35 (17) [0-100] S: 60 (17) [5-100] N: 7 (18) [0-94]
*Light period*			
Males	14.1 (4.2) [6-20]	2097 (413) [1286-2996]	L: 73 (17) [29-100] S: 27 (17) [0-71] N: 0 (0) [0-1]
Females	11.3 (4.2) [3-20]	1499 (393) [173-2467]	L: 44 (20) [0-77] S: 50 (18) [9-90] N: 7 (15) [0-90]
*Transition period*			
Males	3.1 (1.9) [1-10]	72 (38) [10-286]	L: 64 (45) [0-100] S: 36 (45) [0-100] N: 0 (0) [0-4]
Females	2.0 (1.0) [1-5]	52 (28) [0-140]	L: 48 (43) [0-100] S: 45 (41) [0-100] N: 7 (23) [0-100]
*Dark period*			
Males	5.2 (2.3) [1-13]	961 (435) [332-3009]	L: 37 (42) [0-100] S: 63 (41) [0-100] N: 1 (4) [0-36]
Females	4.3 (1.5) [1-9]	730 (279) [71-1489]	L: 20 (23) [0-100] S: 73 (26) [0-100] N: 7 (21) [0-98]

### Area use

The overall average number of zones visited by the birds in a day was 12.77 (SD 4.43). No effects of sex of the birds, side of the house or day of recording were observed on the number of zones visited throughout the day (all *p* > 0.05). Also, when assessing the light and dark periods separately no effects were observed (all *p* > 0.05). However, when assessing the number of zones visited during the transition period, it was observed that male birds visited more zones than female birds (model estimate of 3.02 versus 2.12 on average, *p* = 0.04) and that more zones were visited on the left side of the house (model estimate of 3.04 versus 2.10 on the right side, *p* = 0.03).

### Distances moved

**Fig. 3:**
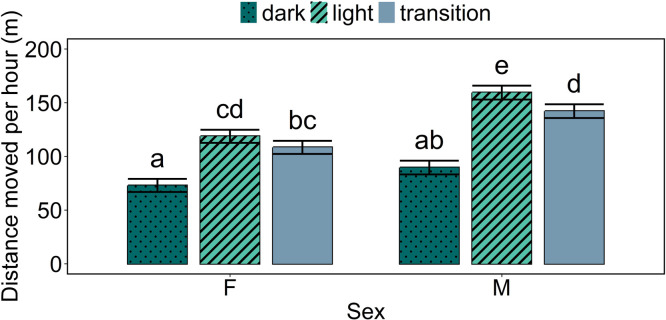
**Model estimated values of the distance moved per hour by sex and period.** Error bars indicate standard errors of the estimated means. Means indicated by a common letter are not significantly different according to the model analysis.

The overall mean total distance moved in a day was 2,679 m (SD 694 m). Regarding total daily distances moved, male birds moved larger distances than female birds (model estimate: 3,098 versus 2,341 m/day; *p* < 0.001) and the distances moved were higher on the left side of the house than on the right side (model estimate: 2,950 versus 2,490 m/day; *p* = 0.02). Regarding distances moved per hour (which were calculated to account for the different durations of the light/dark periods), there was a statistically significant interaction effect between period and sex of the birds (*p* < 0.001; Fig. 3), in which larger distances were walked in the light and transition periods than in the dark period, and male birds furthermore walked longer distances than female birds in the light period and transition period. Within male birds, larger distances were walked in the light period than the transition period, while this difference was not observed in female birds. Moreover, it was again observed that distances moved were higher on the left side of the house than on the right side (model estimate: 129 versus 102 m/hour; *p* = 0.003).

A moderate correlation (tau = 0.27, *p* < 0.001) was observed between the distance moved and the number of zones visited in a day (see **Supplementary data 2** for the correlation plot).

### Time spent in functional zones

**Fig. 4:**
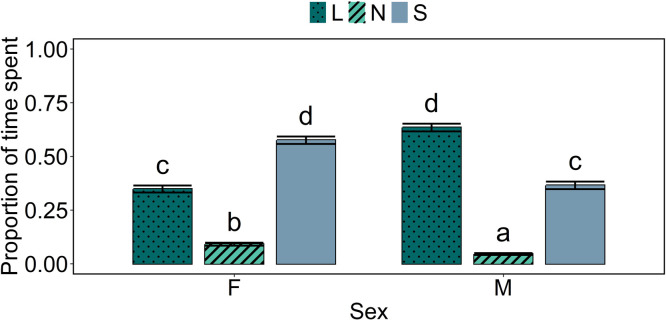
**Estimated proportions of time spent in different functional zones by sex, for full days.** Error bars indicate standard errors of the model-estimated proportions. Means indicated by a common letter are not significantly different according to the beta binomial model analysis. *L* = litter, *N* = nest boxes, *S* = slats.

When looking at full days, there was a statistically significant interaction effect between sex and functional zone in the time spent there (*p* < 0.001; Fig. 4). Male birds spent less time in the nest and slats zones, and more time in the litter zone, than female birds.

**Fig. 5:**
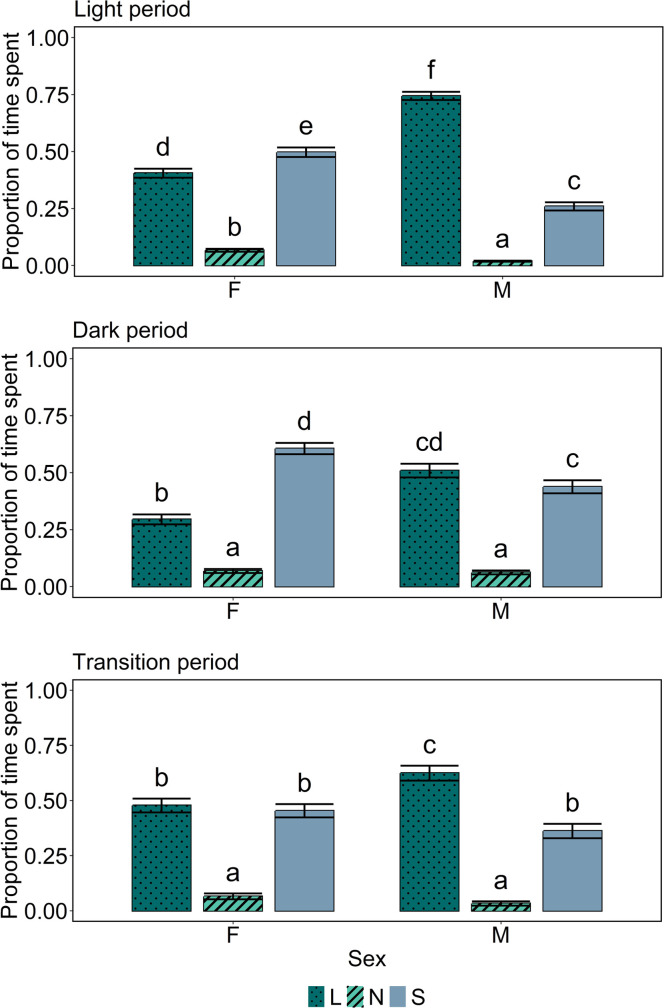
**Estimated proportions of time spent in different functional zones by sex, per period (top: light period; middle: dark period; bottom: transition period).** Error bars indicate standard errors of the estimated proportions. Means indicated by a common letter within a plot are not significantly different according to the beta binomial model analysis. *L* = litter, *N* = nest boxes, *S* = slats.

When looking at the light period, there was a statistically significant interaction effect between sex and functional zone in the time spent there (*p* < 0.001; Fig. 5). Similar as for full days, male birds spent less time in the nest and slats zones, and more time in the litter zone, than female birds. When looking at the dark period, there was also a statistically significant interaction effect between sex and functional zone in the time spent there (*p* < 0.001; Fig. 5). Male birds spent more time in the litter zone, and less time in the slats zone, than female birds. When looking at the transition period, there was again a statistically significant interaction effect between sex and functional zone in the time spent there (*p* < 0.001; Fig. 5). Male birds spent more time in the litter zone than female birds, but there were no statistically significant differences between the sexes for the nest and slats zones.

### Resting location at night

The overall average distance between resting locations at night was 8.98 m (SD 6.77 m). No differences between sexes, days of recording and side of the house were observed in the distance between resting locations at night (all *p* > 0.05).

## Discussion

In this exploratory study it was investigated whether an UWB tracking system could provide insight into broiler breeder activity and spatial behaviour. The tracking data showed that on average broiler breeders used more than half of the area available to them within a day, although this varied between individuals and/or days. It was furthermore observed that - in our small sample of birds - male birds covered larger distances in a day than female birds, and that the distance moved was larger during the light period and transition period than during the dark period. Regarding use of functional areas across the day, it was observed that male birds spent less time in the nest and slats zones, and more time in the litter zone, than female birds. Here, we discuss these observations, as well as general observations and considerations regarding the implementation of UWB tracking in broiler breeder environments.

### Area use

The birds (on average) visited more than half of the area available to them within a day, which is in line with the results of Appleby et al. (1985). However, no effect of sex of the birds was observed on the number of zones visited throughout the day, even though we expected that male birds would use more of the available area than female birds, based on the earlier pilot study performed by our lab (van Emous, unpublished). On the other hand, this observation is in line with Appleby et al. (1985) who observed no differences between the sexes in use of the available area. It is, however, important to note that the type of housing of the birds differed substantially between studies. In the study by Appleby et al. (1985), the birds were housed in a deep litter house. In our earlier pilot study (van Emous, unpublished), the birds were housed in the more typical EU system, which is similar to the house in the current study except for the birds being able to move between the two sides of the house in the pilot study, which was not possible in the current study. Possibly, these differences in housing aspects affect area use by the birds and may result in (absence of) differences in area use between males and females.

However, when specifically focussing on the transition period it was observed that male birds visited more zones than female birds. During the transition period in our study, the lights slowly reduced in intensity. It has been suggested that a reduction in light intensity may serve as a cue to start roosting in chickens (Kent et al., 1997). This roosting may be preceded by a period of increased movement, as in the wild it has been observed that the onset of dark may trigger Red Jungle Fowl to start to move (sometimes distances of 275 m within 15 min) to a roosting place less than half an hour before dark (Collias and Collias, 1967). It can be speculated, given (dominant) males’ role as protectors in the wild (McBride et al., 1969), that males move around more during this time to inspect for potential danger before roosting, but this requires further investigation.

### Distances moved

It was observed that male birds moved larger distances in a day than female birds. Possibly, these higher distances moved by male birds are linked to male birds chasing female birds to mate (Millman et al., 2000), or to male birds trying to monopolize – or mate guard – females, as is observed in the wild (McBride et al., 1969). There may furthermore be differences in distances moved between male birds depending on their ranking, as it has been observed in the wild that the most dominant males have territories to which they restrict their movement, while subordinate males move between multiple neighbouring territories of other groups (McBride et al., 1969).

Within male birds, larger distances were walked in the light period than the transition period, while this difference was not observed in female birds. Earlier studies have shown that mating behaviour is most frequent later in the light period (e.g., Duncan et al., 1990; Bilcik and Estevez, 2005). However, it may be speculated that males reduce their mating efforts when it starts to go dark, when in the wild they start moving towards a place to roost (Collias and Collias, 1967). This might explain the lower observed activity levels in the transition period than in the light period.

In general, distances moved were lower during the dark period than during the light period and transition period, which was expected given the observed circadian rhythms of chickens (see e.g. Abecia et al. (2025) who studied locomotor activity in laying hens) and the general low activity of chickens during the dark. For example, in broilers it has been observed that approximately 98% of the dark period was spent resting or sleeping (Deep et al., 2012). Nonetheless, it is important to note that some movement was also recorded during the night, as the distances moved in the dark period were not zero. In the study by Deep et al. (2012), approximately 2% of the dark period was spent on active behaviours (including standing, walking, running, feeding, drinking, foraging, preening, dustbathing, stretching, feather ruffling and wing-flapping). However, in the current study we hypothesize that the recorded distances at night might be largely due to noise in the UWB system (see further on).

### Time spent in functional zones

For full days, as well as the light period, male birds spent less time in the nest and slats zones, and more time in the litter zone, than female birds. This is as expected, given that the feed for the males was located in the litter zone and that most matings are also expected to take place on the litter (van Emous, 2023). The females, on the other hand, generally need to visit the nest boxes and their feed was partially located above the slats. A similar pattern was observed in the transition period and dark period, with male birds spending more time in the litter zone than female birds, but no differences between the sexes were observed in the time spent in the nest box zone during these time periods. This is likely caused by the nest boxes remaining closed during the transition period and most of the night and female birds therefore not being able to access the nests.

### Resting location at night

Even though the descriptive statistics indicated individual variation between birds and/or days in their preferred range of resting locations, with observed ranges from 0.3 to 43.1 m distance between subsequent resting locations for the night, no differences between the sexes were observed. This suggests that male and female birds do not differ consistently in their preference for the range of their preferred resting locations.

### Using UWB to track broiler breeders

Overall, the implementation of an UWB tracking system to record activity and area use in broiler breeders was considered successful. However, the use of an UWB system in a commercial broiler breeder house comes with some challenges. Several broiler breeders (25%) lost their UWB backpacks, resulting in fewer birds with complete data available than initially fitted with an UWB tag. These losses of backpacks might have been linked to the size of the birds, as the backpacks could more easily come loose in smaller birds. This may have especially been the case in female birds, as 5 out of 12 female birds lost their backpack, while only one out of the total of 8 male birds lost its backpack. In future studies, differently sized backpacks could potentially be used for male and female birds or tighter elastic bands around the wing base could be used to avoid loss of tags.

In this study, we did not compare behaviour and performance of tagged birds and birds without UWB backpacks. However, after taking off the backpacks at the end of the study, we observed no physical damage to the birds and even some better feather quality on the females, likely due to the backpack providing protection from scratching by the males during mating (*personal observation*). In studies in which backpacks with UWB tags were fitted to slow- or fast-growing broilers, little persistent impact on the birds’ behaviour, leg health or performance was observed (Stadig et al., 2018b; Baxter and O’Connell, 2020). The birds in our study already wore the UWB backpacks for at least a full day before the data analysis started, so it is reasonable to assume that the birds were largely used to their backpacks at this point.

Another challenge is that poultry housing systems contain a lot of (metal) equipment, as well as large flocks of birds, and this may cause some interference to the UWB signal. Stadig et al. (2018a) studied the accuracy and registration success of an UWB system in a free-range area. In terms of accuracy (median error of the location estimate), they observed that in some instances the errors were larger when tags were placed on chickens as opposed to when no chickens were present, but observed no clear impact of different vegetation types, rainy days, other tags being present in the proximity or not, the height and placement of the tag, the angle of the tag, the orientation of the tag, and of coverage of the tags by a mobile house, cardboard box or wooden frame. In this study, we sometimes observed recorded detections on the other side of the house than the bird was kept in, i.e., in locations where the bird could not be. However, this was the case for only a small subset of the recorded detections (0.015%). Moreover, birds were sometimes detected outside the range of the house or pen, but this was the case for only approximately 1.8% of the detections according to our calculations (these detections outside the range of the house were reclassified into the nearest zone, see methods section). It must, however, be noted that it might have been slightly more in reality, as we determined these erroneous detections based on the outer coordinates of the zones, while zones 1, 21 and 31 are not rectangular (Fig. 2). In a small pilot study (data not shown), we placed two (mostly) stationary tags in the poultry house and assessed their recorded position over time. One tag was placed on the underside of the slats (fully stationary), while the other tag was placed on the drinking line (which could swing back and forth a bit when birds pushed the drinker line). Looking at a period of 25 days, average hourly distances moved of 0.8 m (range: 0 – 12.7) and 4.2 m (range: 0 – 28.3) were observed, for the fully stationary tag and the tag on the drinking line respectively. This shows that there is some noise in the location detection, which may add up over time to overestimated distances moved. Other studies have indeed observed that their respective UWB systems overestimated the distances moved by broilers. Van der Sluis et al. (2019) implemented an UWB system for fast-growing broilers, using a sampling rate of one sample every seven seconds approximately, and observed that the UWB system on average overestimated the true distance moved by 10%. Baxter and O’Connell (2020) observed an average overestimation of 144% in their study, using a sampling rate of 100 milliseconds. Nonetheless, the authors of both these studies concluded, based on the correlation between UWB-recorded distances and true distances, that the UWB systems are able to provide insight into relative distances moved. Overall, with UWB systems, we can obtain insight into individual differences in activity and area use, allowing for longitudinal and continuous data collection.

### Translation to practice

The individual movement patterns of broiler breeders, as obtained using an UWB system, can provide insight into birds’ preferred locations and subsequently inform housing design and bird management based on the observed preferences. It is, however, important to note that the insights obtained in this study were derived from only a small subset of birds (*n* = 15), especially when one takes into account that these birds were divided over two halves of the house, and a short period of time (3 weeks). In this study, we observed use of a larger area during the transition period and overall higher distances moved on the left side of the house than on the right side. Possibly, this is related to the main entrance to the house being located on the left-hand side of the house, indicating some potential disturbance of the birds when people enter the house. However, given the small sample size per side of the house, this would require further investigation. For future work, it is recommended to also assess birds in other (types of) houses, to determine whether the observations made in this study generalize across different environments. Furthermore, it is recommended to study a larger subset of birds and for a longer period, to provide more insight into individual variation and consistency in movement patterns over time. If more data is collected on the selected birds themselves (e.g. health or performance data, such as feather cover or mating activity), this can provide a starting point for studying the potential use of movement patterns as a proxy for bird performance.

## Conclusion

In summary, the tracking data showed that on average broiler breeders used more than half of the area available to them within a day, although this varied between individuals and/or days. Male birds covered larger distances in a day than female birds, and the distance moved was larger during the light period and transition period than during the dark period. It was observed that male birds spent less time in the nest and slats zones, and more time in the litter zone, than female birds. The overall average distance between resting locations at night was 8.98 (SD 6.77) m, and no differences were observed in this between the sexes. Overall, although the sample size was very limited and our findings should therefore be considered as preliminary, these observations do offer a first step towards providing insight into broiler breeders’ movement patterns and preferred locations, which can in the future inform housing design and bird management.

## CRediT authorship contribution statement

**Malou van der Sluis:** Writing – review & editing, Writing – original draft, Visualization, Formal analysis. **Annemarie J.W. Mens:** Writing – review & editing, Writing – original draft, Investigation. **Rick A. van Emous:** Writing – review & editing, Project administration, Investigation, Funding acquisition, Conceptualization.

## Disclosures

The authors declare that they have no known competing financial interests or personal relationships that could have appeared to influence the work reported in this paper.

## Appendix Supplementary materials

Image, application 1

Image, application 2
