# Supplementary material for: Automated tracking of broiler breeder activity and functional area use in a commercial housing system: differences between sexes and time of day
Source: Poult Sci. 2025 Dec 18;105(2):106304. doi: 10.1016/j.psj.2025.106304 (PMC12800487; doi:10.1016/j.psj.2025.106304)
Supplement: Supplementary file 1 [file mmc1.docx]

**Supplementary Data 1: Number of detections on the wrong side of the pen**

| ID | Side bird is housed | L1 | S1 | N1 | N2 | S2 | L2 | Total | Wrong side | % wrong |
| --- | --- | --- | --- | --- | --- | --- | --- | --- | --- | --- |
| 1 | 2 | 0 | 1 | 243 | 278192 | 430888 | 68713 | 778037 | 244 | 0.031% |
| 2 | 2 | 0 | 3 | 9 | 107 | 694292 | 358668 | 1053079 | 12 | 0.001% |
| 3 | 1 | 962027 | 37891 | 113 | 0 | 0 | 0 | 1000031 | 0 | 0% |
| 4 | 2 | 0 | 0 | 6 | 5674 | 464102 | 337297 | 807079 | 6 | 0.001% |
| 5 | 2 | 0 | 9 | 38 | 654 | 552869 | 494836 | 1048406 | 47 | 0.004% |
| 6 | 1 | 389324 | 643746 | 2548 | 215 | 0 | 0 | 1035633 | 215 | 0.021% |
| 7 | 2 | 0 | 0 | 33 | 28805 | 433140 | 538838 | 1000816 | 33 | 0.003% |
| 8 | 2 | 0 | 0 | 0 | 4657 | 660141 | 246830 | 911628 | 0 | 0% |
| 9 | 1 | 413182 | 659553 | 1236 | 0 | 0 | 0 | 1073971 | 0 | 0% |
| 10 | 1 | 333751 | 529883 | 7618 | 159 | 6 | 0 | 871417 | 165 | 0.019% |
| 11 | 2 | 0 | 0 | 28 | 10796 | 527272 | 395637 | 933733 | 28 | 0.003% |
| 12 | 1 | 289165 | 689555 | 13587 | 146 | 2 | 0 | 992455 | 148 | 0.015% |
| 13 | 1 | 557560 | 427031 | 20952 | 533 | 109 | 0 | 1006185 | 642 | 0.064% |
| 14 | 1 | 266212 | 593743 | 40270 | 613 | 42 | 0 | 900880 | 655 | 0.073% |
| 15 | 2 | 0 | 0 | 0 | 42 | 110931 | 930377 | 1041350 | 0 | 0% |
| Total |  |  |  |  |  |  |  | 14454700 | 2195 | 0.015% |

L = litter area; S = slatted area; N = nest boxes. Sides are indicated with numbers 1 and 2, e.g. L1 = litter area on side 1.
