# Supplementary material for: Automated tracking of broiler breeder activity and functional area use in a commercial housing system: differences between sexes and time of day
Source: Poult Sci. 2025 Dec 18;105(2):106304. doi: 10.1016/j.psj.2025.106304 (PMC12800487; doi:10.1016/j.psj.2025.106304)
Supplement: Supplementary file 2 [file mmc2.docx]

**Supplementary Data 2: Plot of the correlation between distances moved and zones visited**


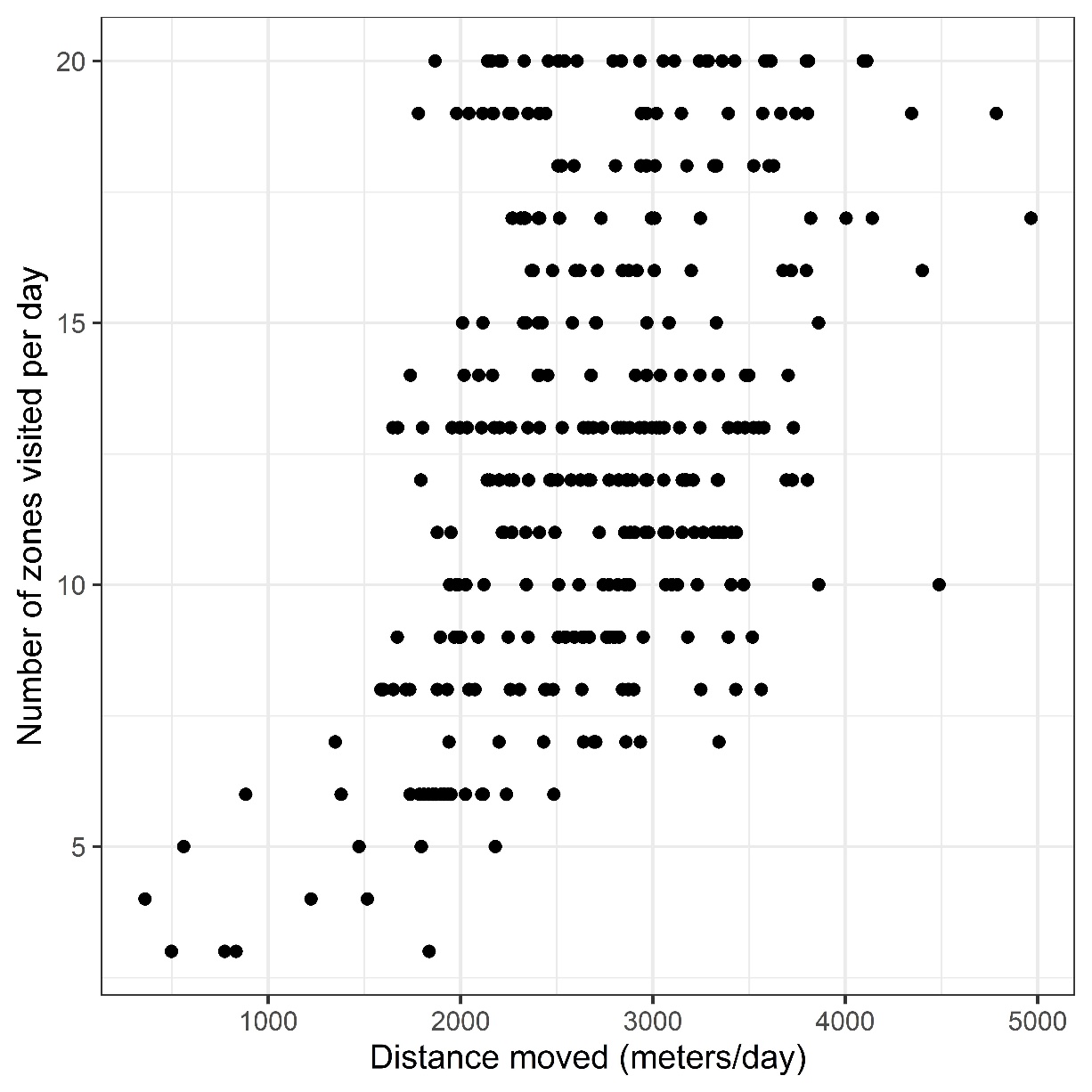


Figure S2.1: Plot of the correlation between the distance moved and number of zones visited per day.
